# Short Peptides Make a Big Difference: The Role of Botany-Derived AMPs in Disease Control and Protection of Human Health

**DOI:** 10.3390/ijms222111363

**Published:** 2021-10-21

**Authors:** Xiumei Luo, Wenxian Wu, Li Feng, Haim Treves, Maozhi Ren

**Affiliations:** 1 Institute of Urban Agriculture, Chinese Academy of Agricultural Sciences, Chengdu Agricultural Science and Technology Center, Chengdu 610000, China; luoxiumei@caas.cn (X.L.); wuwenxian@caas.cn (W.W.); fengli@caas.cn (L.F.); 2 Key Laboratory of Plant Hormones and Development Regulation of Chongqing, School of Life Sciences, Chongqing University, Chongqing 401331, China; 3 Zhengzhou Research Base, State Key Laboratory of Cotton Biology, School of Agricultural Science of Zhengzhou University, Zhengzhou 450000, China; 4 School of Plant Sciences and Food Security, Tel-Aviv University, Tel-Aviv 69978, Israel; htreves@tauex.tau.ac.il; 5 Hainan Yazhou Bay Seed Laboratory, Sanya 572025, China

**Keywords:** botany-derived antimicrobial peptides, mechanism of action, molecular targets, disease prevention and control, health security

## Abstract

Botany-derived antimicrobial peptides (BAMPs), a class of small, cysteine-rich peptides produced in plants, are an important component of the plant immune system. Both in vivo and in vitro experiments have demonstrated their powerful antimicrobial activity. Besides in plants, BAMPs have cross-kingdom applications in human health, with toxic and/or inhibitory effects against a variety of tumor cells and viruses. With their diverse molecular structures, broad-spectrum antimicrobial activity, multiple mechanisms of action, and low cytotoxicity, BAMPs provide ideal backbones for drug design, and are potential candidates for plant protection and disease treatment. Lots of original research has elucidated the properties and antimicrobial mechanisms of BAMPs, and characterized their surface receptors and in vivo targets in pathogens. In this paper, we review and introduce five kinds of representative BAMPs belonging to the pathogenesis-related protein family, dissect their antifungal, antiviral, and anticancer mechanisms, and forecast their prospects in agriculture and global human health. Through the deeper understanding of BAMPs, we provide novel insights for their applications in broad-spectrum and durable plant disease prevention and control, and an outlook on the use of BAMPs in anticancer and antiviral drug design.

## 1. Introduction

Pathogenic microorganisms pose a great threat to plants, animals, and humans. Global crop losses caused by bacteria, fungi, and viruses amount to USD 220 billion annually. Humans have also fought against pathogenic microorganisms throughout history. Catastrophic events such as smallpox, plague, and influenza change the course of human development time and time again. At present, we are still suffering from the devastating influence of SARS-CoV-2. During the “arms race” against pathogens, all organisms have evolved strategies to survive pathogenic infections. Among them, plants have developed unique, multi-level defense mechanisms against pathogens, including the use of physical barriers in the cell wall, induction of hypersensitive defense responses, expression of resistance proteins, and synthesis of antimicrobial peptides (AMPs) [1]. Botany-derived AMPs (BAMPs), a class of ubiquitous cationic polypeptides with less than 10 kDa of molecular weight, are the first line of defense in the non-specific innate immune system of plants [2]. Composed of 20–60 amino acid residues, BAMPs are characterized by strong basicity, thermal stability, and broad-spectrum antimicrobial activity. Unlike the well-known PTI (pattern-associated triggered immunity) and ETI (effector-associated triggered immunity) immune responses in plants, the broad-spectrum antimicrobial activity of BAMPs is largely due to targeting lipid structures of microbial cell membranes, thus disrupting membrane structure [3,4] and leading to content leakage and cell death through a combination of membrane lysis and cytotoxicity [5,6]. However, more complex mechanisms also exist, including interaction with specific lipids, cell cycle arrest, reactive oxygen species (ROS) production, programmed cell death, autophagy, cell signaling, and immune response [7,8]. BAMPs are thus responsible for durable resistance in plants.

Since the first BAMP was isolated from wheat, 352 BAMPs have been identified in different plant species [9], which has greatly enriched our knowledge of BAMPs. Additionally, the structures of more than 70 BAMPs have been elucidated. These BAMPs not only exhibit antimicrobial activity in plants, but some also exert anticancer and antiviral effects in humans. They are mainly classified as those with typical structures rich in cysteine residues (such as cyclotides, defensins, thionins, lipid transfer proteins (LTPs), snakins, hevein-like, and knottin-like), and those with atypical structures with few/no cysteine residues (such as 2S albumin, hairpinins, puroindolines, myrosinase binding protein, glycine-rich peptides, αβ-trumpet, and short non-disulfide peptides) [10]. The amino acid composition and structure of BAMPs vary greatly among different classes, usually forming multiple pairs of disulfide bonds that confer structural and thermodynamic stability. Their common features include: (a) ribosome-derived secretory proteins, and the precursor consists of a signal peptide at the N-terminus, a mature AMP structural domain, and an acidic domain at the C-terminus, (b) small molecular weight, (c) cysteine-rich, forming 2–6 intramolecular S-S bonds, and (d) compact structure, conferring thermal, chemical, and enzymatic stability [11]. BAMPs play an important role in regulating plant growth and development, as well as responses to abiotic stress (drought, cold, salt, injury). Moreover, they are highly induced under biotic stress, especially upon pathogen infection [12]. In the field of plant protection, great progress has been made in improving disease resistance in crops by inducing homologous or heterologous expression of BAMPs. For example, heterologous expression of radish defensin RsAFP2 in tobacco, tomato, and wheat significantly improves crop resistance to fungi [13,14], while expression of petunia defensins PhDef1 and PhDef2 increases protection against *Fusarium oxysporum* in banana crops [15]. In the medical field, BAMPs have been found to significantly inhibit the proliferation of cancer cells and viruses, such as the nonspecific lipid transfer protein (NTP) isolated from *Narcissus tazetta* [16]. At present, the direct and prevalent mechanism underlying the microbicidal effects of BAMPs is strengthening the membrane permeability, thus leading to metabolite leakage and ultimately cell death [1,8,17]. Additionally, BAMPs play an indirect microbicidal role by targeting organelles, nucleic acids, proteins, and cascade signaling pathways in pathogens after internalization through cell surface receptors or active permeation [1,8,17].

The classification of BAMPs, their structure–function relationships, and research progress in plant development and host defense have been thoroughly described previously (see reviews in [1,8,17]). This review will focus on the properties and functions of BAMPs belonging to the pathogenesis-related (PR) protein family, antimicrobial mechanisms of BAMPs, potential links between membrane components (lipids and membrane proteins), and mechanisms of action, intracellular targets, and potential applications of BAMPs in plant disease control and human disease treatment. This review deepens our understanding of BAMPs in plant protection and human health, and provides theoretical input for future research on BAMPs, thus enabling the development of new disease control strategies and therapeutics.

## 2. BAMP Diversity and Mechanisms of Action in Plants

In this section, we will describe the antimicrobial activity and antimicrobial mechanisms of BAMPs belonging to the PR family (Table 1). Since they can be cleaved to form mature active peptides that inhibit pathogens, the PR1 and protease inhibitor PR6 with low molecular weight and cysteine-rich are also categorized as BAMPs and reviewed in this section.

### 2.1. Plant Defensins: Interacting with Fungal Membrane Components and Targeting Intracellular Pathways

**Figure 1:**
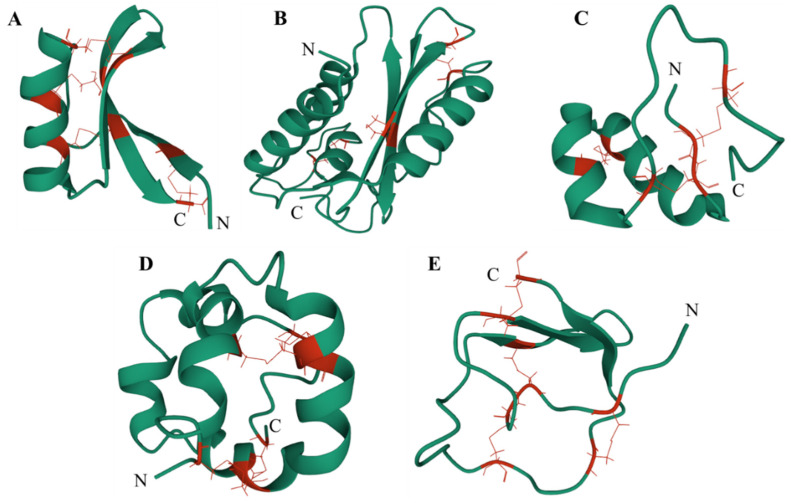
3D structures of BAMPs. (**A**) Plant defensin from Raphanus sativus (RsAFP2; PDB: 2N2R; Accession: 2N2R_A; pI: 8.70). (**B**) PR1 from Solanum lycopersicum (P14A; PDB: 1CFE; Accession: 1CFE_A; pI: 8.94). (**C**) Thionin from Viscum album (Viscotoxin B; PDB: 1JMP; Accession: 1JMP_A; pI: 8.77). (**D**) Lipid transfer protein from Oryza sativa (nsLTP2; PDB: 1L6H; Accession: 1L6H_A; pI: 8.72). (**E**) Proteinase-inhibitor from Capsicum annum (HyPep; PDB: 5ZFO; Accession: 5ZFO_A; pI: 6.15). N and C represent N-terminus and C-terminus, respectively. Green represents the amino acid skeleton. Red represents cysteines and disulfide bonds between cysteines.

Plant defensins, which belong to the PR12 family, are basic BAMPs consisting of 45–54 amino acid residues and four disulfide bonds with less than 5 kDa of molecular weight (Figure 1A) [18]. Their antimicrobial activity is mainly directed against fungi and oomycetes, with relatively few effects on bacteria [12]. Since initial isolation from barley and wheat endosperm in 1990, defensins have been subsequently isolated and identified from different tissues of various monocotyledonous and dicotyledonous plants [19]. Moreover, defensins are widely present in insects and animals for protection against invasion by bacteria, fungi, or viruses, and are an important component of the immune response system. Plant defensins can be broadly classified into classes I and II based on the structure of their precursor proteins. Class I defensins contain endoplasmic reticulum (ER) signaling sequences and mature defensin domains, while class II defensins contain a C-terminal prepropeptide (CTPP) of 27–33 amino acid residues in addition to the characteristics of class I defensins. They are usually produced in solanaceous species and constitutively expressed in flowers and fruits [12]. Class I defensins enter the secretory pathway directly upon synthesis. They lack the signal sequences for post-translational modification or subcellular targeting, and accumulate in the cell wall and extracellular space [19]. With a CTPP that targets vesicles, class II defensins mostly undergo proteolysis in the vesicle to release mature short peptides [12,20]. Mature defensins consist of five segments of non-conserved loops, linking α-helices and β-strands to form high-level structures. Differences in the loop sequences confer different functions, including inhibition of protein synthesis, antimicrobial activity, heavy metal tolerance, plant development, and blocking of ion channels [21].

The amphiphilic characteristics of defensins allow them to bind specifically to the sphingolipid or phospholipid structure of the pathogenic fungal plasma membrane, and preferentially to lipid II [22,23], sphingolipid mannosyldiinositolphosphorylceramide (M(IP)2C) [24], glycosylceramide (GlcCer) [25], phosphatidic acid (PA) [26], and phosphatidylinositol-4,5-bisphosphate (PI(4,5)P2) [27]. Their binding specificity is usually mediated by the loop 5 region [21,22]. For example, defensins RsAFP2 in radish and DmAMP1 in dahlia specifically bind to GlcCer and M(IP)2C on the fungal membrane, respectively. The binding of DmAMP1 to M(IP)2C triggers rapid fungal responses that include increased Ca^2+^ uptake and K^+^ efflux, irreversible changes in membrane permeability, and activation of the fungal cell wall integrity (CWI) pathway [25,28,29]. The interaction of RsAFP2 and MsDef1 with GlcCer in the cell wall and plasma membrane induces ROS production, followed by damaging proteins, lipids, and DNA, and activating apoptosis or programmed cell death [30]. Further, RsAFP2 and MsDef1 act as signaling molecules, activating the mitogen-activated protein kinase (MAPK) signaling cascade response [31,32,33].

However, some defensins, such as MtDef4, NaD1, Psd1, and HsAFP1, require internalization into cells to function. These peptides enter the cytoplasm in different ways, including energy-dependent endocytosis, polyamine transport systems, and passive transport. MtDef4 from *Tribulus terrestris* is internalized into fungal cells via binding to PA, exerting an antimicrobial effect in vivo by disrupting Ca^2+^ homeostasis and interacting with unknown intracellular targets [34]. NaD1, a class II defensin produced in the flowers of ornamental tobacco, protects reproductive tissues against damage by fungal pathogens. NaD1 requires the presence of a cell wall to initiate its specific lethal effect on fungal cells, where it binds tightly to PI(4,5)P2 to form a dimer before being translocated to the cytoplasm and interacting with intracellular targets to trigger ROS and nitric oxide (NO) production, permeabilization of the plasma membrane, granulation of the cytoplasm, and cell death [33,35]. HsAFP1, an antimicrobial peptide isolated from the seeds of *Hemerocallis pigmenti*, binds to the fungal cell wall and plasma membrane via loop 5, penetrates the cytoplasm after internalization into pathogen cells, targets mitochondria, inhibits the respiratory chain, produces ROS, and induces apoptosis, thus leading to pathogenic cell death [36]. Interestingly, defensins such as Psd1 from *Pisum sativum* can also enter the nucleus via nuclear migration to target and inhibit cyclin F, leading to cell cycle arrest and cell death [37].

The various interaction mechanisms between plant defensins and pathogens explain why these short peptides have contributed to innate plant immunity as potent antimicrobial molecules for thousands of years, and why they are attractive antimicrobial drug candidates for agricultural and clinical use. Although the mechanism by which plant defensins inhibit pathogen growth at the plasma membrane level through specific binding to sphingolipids (phospholipids) on the plasma membrane has been demonstrated and generally accepted, little is known about the internalization mechanism and the mode of action within pathogens.

### 2.2. CAPE Peptides (PR1): Binding to Sterols on Pathogen Membrane and Inhibiting Programmed Cell Death

PR1 is a small, secreted or vesicle-targeting antimicrobial protein specifically induced by salicylic acid (SA) [38]. The mature protein contains approximately six conserved cysteine residues, forming a tertiary α-β-α structure through three pairs of disulfide bonds (Figure 1B). As a marker protein for PTI and systemic acquired resistance (SAR), PR1 provides broad-spectrum resistance to a wide range of pathogens [39,40]. PR1 is also found in yeast, insects, and vertebrates, including humans, which form the cysteine-rich secretory protein (CAP) superfamily, together with cysteine-rich secretory proteins (CRISPs) and antigen 5. Although PR1 is one of the most abundant proteins in the apoplast during pathogenic infection, little is known about its mechanism of action [18]. The role of PR1 in plant–pathogen interactions remains unclear.

PR1a from tobacco was the first PR1 antimicrobial protein to be identified. Overexpression of PR1a significantly increases the tolerance of *Nicotiana tabacum* to tobacco downy mildew (*Peronospora tabacina*) and black shank (*Phytophthora parasitica* var. *nicotianae*) [41]. The synergistic overexpression of PR1a with P14c from tomato significantly inhibits the germination of *Phytophthora* *infestans* spores and suppresses its colonization in the host [42]. *P. infestans* is a sterol-auxotrophic pathogen that is highly sensitive to PR1 because PR1 sequesters sterols from the pathogen membrane, thus inhibiting pathogen growth [43]. Additionally, sterol-prototrophic pathogenic fungi exhibit high sensitivity to PR1 when their sterol biosynthesis is blocked, suggesting a positive correlation between the sterol-binding capacity of PR1 and its antifungal properties, and that the antimicrobial action of PR1 depends on the sterol synthesis ability of different microorganisms [43]. Further study demonstrated that PR1 bind sterols through its conserved CAP structural domain, which is essential for its antimicrobial activity and is conserved in the CAP superfamily and pathogen-related yeast (PRY) proteins [44].

In addition to sequestering sterols, PR1 can also inhibit programmed cell death upon pathogen infection [45] and induce the expression of host defense-related genes by releasing CAPE1 (CAP-derived peptide 1), a defense signal peptide. CAPE1, with the conserved motif PxGNxxxxxPY, originally derived from the last 11 amino acid residues of the C-terminus of tomato P14c, and is later cleaved at the C-terminus of PR1 and PR5 in a variety of plants [46,47]. CAPE1 generates resistance to *Pseudomonas syringae* DC3000 and the larvae of *Spodoptera litura* by inducing the expression of defense-related genes, and induces immune responses against herbivores, pathogens, and abiotic stress [46,47]. These data suggest that CAPE1 plays an important role in inducing host immune responses. Significant progress has been made in our understanding of PR1 function since CAPE1 peptides were identified and their roles in biotic stress responses were established.

Moreover, an increasing number of studies have shown that PR1 promotes cell death by interacting with a variety of pathogen effectors [48]. For example, the ToxA effector of wheat pathogens *Parastagonospora nodorum* and *Pyrenophora tritici-repentis* interacts with the dimeric TaPR1-5 protein, leading to increased host cell necrosis [48]. Additionally, the *P. nodorum* effector SnTox3 interacts with multiple wheat PR1 family members [49] and prevents TaPR1 from releasing the TaCAPE1 peptide [40]. Furthermore, the important virulence factor SsCP1 in *Sclerotinia sclerotiorum* targets PR1 in *Arabidopsis thaliana* to inhibit its function, but SsCP1 can also be recognized by the plant, which triggers defense responses that lead to compatible interaction [50]. Interactions between PR1 and pathogen effectors suggest that when pathogens invade susceptible host plants, they secrete effectors to target PR1, thereby inhibiting its antimicrobial activity and achieving pathogen colonization and infection in the host. Conversely, resistant host plants express and secrete PR1 to target pathogen effectors or key virulence proteins associated with pathogenicity when invaded by pathogens, thereby achieving antimicrobial activity to control disease.

### 2.3. Thionins (PR13): Binding to Phospholipids and Disrupting Membrane Permeability

Thionins are a class of small molecular weight (5 kDa), cysteine-rich, cytotoxic, basic BAMPs that belong to the PR13 family [51]. They contain 6 or 8 cysteines, and 3 or 4 disulfide bonds (Figure 1C). Originally isolated from cereals, approximately 100 thionins have since been identified in 15 monocotyledonous and dicotyledonous plant species [52]. Thionins are divided into two families with different structures, origins, and functions: α/β-thionins and γ-thionins. α/β-thionins are true PR13 family members, whereas γ-thionins are in common with defensins and belong to the PR12 family [53]. α/β-thionins have high sequence similarity with 45–48 amino acid residues and 3–4 disulfide bonds, and can be classified into types I–V [51]. Except for type IV thionins, which are neutral peptides, the other types of thionins are basic, with type V thionins consisting of a class of truncated peptides without cytotoxicity. The N-terminus of the thionin precursor protein contains a leading peptide of about 20 amino acid residues, while the C-terminus contains an acidic peptide of about 60 amino acid residues to neutralize the cationic bioactive peptide prior to its final maturation stage. Cleavage of the leading peptide is required for its toxic activity, and the mature domain is less conserved than the two flanking domains [54].

Thionins play an important role in the defense against pathogen invasion, with overexpression of thionin-encoding genes increasing disease resistance. For example, overexpression of endogenous *Thi2.1* enhances the resistance of *A. thaliana* to *F. oxysporum* [55], while heterologous overexpression of *Thi2.1* from *A. thaliana* in tomato results in enhanced resistance to bacterial wilt and blight [56]. Further, high-level expression of the *hordothionin* gene from barley in tobacco confers resistance to *Pseudomonas syringae* [57], while rice plants expressing the oat *thionin* gene display enhanced resistance to bacterial diseases [58]. Various studies have confirmed that thionins can act directly on cell membranes and exert antimicrobial activity by altering membrane permeability or forming ion channels [52]. Positively charged thionins have a strong electrostatic binding capacity to negatively charged phospholipids on the microbial membrane, which are naturally present as a protein–lipid complex [59]. In the resistance to pathogen invasion, thionins disrupt pathogenic membrane permeability in a dose-dependent manner, with a critical dose (approximately 1 μM) directly associated with antimicrobial activity and membrane lysis, and an approximately 1 h duration of activity [60]. In addition, thionins interact with lipid transfer proteins (LTPs) to exert synergistic antimicrobial activity, suggesting that these proteins may cooperate in membrane binding and/or permeation [52,61].

Besides cytotoxicity, thionins are involved in other cellular processes. Experiments performed on artificial cell membranes and different cell lines demonstrated that thionin treatment depolarizes membranes, causes cell lysis, and increases Ca^2+^ channel permeability [62]. After binding to calmodulin, thionins activate endogenous phospholipase A2 (PLA2) and adenylate cyclase, inhibit protein kinase C, and suppress DNA and protein synthesis in cell-free systems [52]. Furthermore, thionins possess thioredoxin activity and are involved in redox regulation of enzymes as a secondary messenger [62]. These events following cell membrane disruption amplify the initial toxic effects of thionins and disrupt many key cellular processes, ultimately leading to cell death [63]. Overall, thionins directly target negatively charged phospholipids in the cell membrane rather than specific protein receptors on the cell surface [52]. Any membrane containing neutral or moderately cationic phospholipids can also be disrupted by thionins [64].

### 2.4. Lipid Transfer Proteins (LTPs, PR14): Nonspecifically Transporting Lipids

LTPs are a class of multigene-encoded, abundant, soluble, and structurally compact cationic small peptides [65]. As the name implies, the primary function of LTPs is to facilitate the transfer of various types of lipids, including phosphatidylinositol, phosphatidylcholine, and galactolipids [66]. With their low specificity for lipid substrates, plant LTPs are also known as nonspecific lipid transfer proteins (nLTPs). Their protein structure contains four conserved disulfide bonds, and 4–5 α-helices fold to form a tight, heat- and denaturant-insensitive three-dimensional structure (Figure 1D). Hydrophobic cavities are formed to facilitate lipid binding and transport [67]. Based on the spacing between cysteine residues, sequence differences, and post-translational modifications, LTPs are divided into five major classes (LTP1, LTP2, LTPc, LTPd, LTPg) and five minor classes (LTPe, LTPf, LTPh, LTPj, LTPK) [68]. Among the major classes, LTPd and LTPg are present in all terrestrial plants, suggesting that they may be the earliest evolved LTPs, whereas LTP1 and LTP2, the most well-studied LTPs in flowering plants, likely evolved later and are not found in algae, mosses, or other non-seed plants [68]. The N-terminus of the LTP precursor contains a signal peptide for the cellular secretion pathway that locates LTPs in the intercellular space outside the plasma membrane [69]. LTPs have a variety of biological activities, such as promoting cell expansion and plant growth [70], participating in lipid metabolism [71], and being responsible for wax and lipid barrier polymer deposition [72,73,74]. In addition, LTPs are an important part of plant defense, with their encoding genes being abundantly expressed in response to pathogen infection. Furthermore, transgenic overexpression of *LTP* genes enhances host tolerance to pathogen infection; thus, LTPs are classified as members of the PR14 protein family. Homologous overexpression of *LTP* genes and heterologous overexpression of barley *LTP* genes in *A. thaliana* enhance plant tolerance to *P. syringae* and *Botrytis cinerea* [75], while the expression of barley *LTP* genes in tobacco also enhances its resistance to *P. syringae* [76]. CaLTP1 isolated from *Capsicum annuum* seeds exerts antimicrobial activity against *Saccharomyces cerevisiae*, *Pseudomonas tropicalis*, and *Colletotrichum* *lindemuthianum*, causing morphological damage by penetrating the plasma, and leading to pseudo-hyphae formation [77]. All four nsLTP homologous peptides (CW18–21) isolated from barley and maize can inhibit *Clavibacter michiganensis*, *P. solanacearum*, and *F. solani* [61]. Other LTPs, such as Ha-AP10 [78], Ace-AMP1 [79], and NTP [61,80], can inhibit the growth, development, and pathogenicity of pathogenic fungi and bacteria to varying degrees.

LTPs in plants exhibit broad-spectrum antimicrobial activity by inhibiting the growth of pathogens, and exhibit low toxicity to plant and mammalian cells [81]. However, little has been reported on their antimicrobial mechanism. It has been hypothesized that LTPs may interact with lipids in microbial membranes, causing the lipids to translocate to the extracellular compartment, thus leading to membrane permeation or apoptosis [82]. The detailed mechanism of action can be found in the model of LTPs associated with carcinogenesis (see Section 3.3).

### 2.5. Proteinase-Inhibitor (PIs, PR6): Inhibiting Proteinase Activity That Is Essential for Pathogen Growth and Pathogenicity

PIs, which belong to the PR6 family, are a subclass of tomato/potato inhibitor I-related serine PIs, with a molecular weight of 8 kDa and 4 disulfide bonds (Figure 1E) [83,84]. Since all types of PIs can interact with pathogenic proteases to exert host defense functions, limiting PIs to serine protease inhibitors is controversial [85,86]. PIs have multiple biological functions, including regulation of endogenous proteases during seed dormancy, mobilization of protein reserves [86], and host defense [87]. During disease defense, PIs can reduce pathogen aggressiveness by inhibiting the lyase activity required for fungal pathogenicity [88], blocking the replication cycle of viruses [89], and inhibiting the digestive enzyme activity of nematodes and insects, thereby limiting the release of amino acids [90]. Both fungal and bacterial infections can induce substantial expression of *PI* genes. For example, *P. infestans* [91] and *P. syringae* [92] induce the expression of *PI* genes in tomato. In vitro experiments have shown that barley trypsin PI could inhibit *Alternaria brassicola*, *Ascochyta pisi*, *F. culmorum*, and *Verticillium dahliae* [93], and the activity is synergistically enhanced when combined with thionin (PR13). In addition, HyPep, a serine PI isolated from *C. annuum* seeds, is able to completely inhibit the growth of *S. cerevisiae* and *C. tropicalis* at 25 mg/mL, leading to cell aggregation and pseudo-hyphae formation [94]. Buckwheat trypsin PI is able to inhibit the protease activity necessary for the pathogenicity of *B. cinerea* in vitro [88,95]. Lorito et al. [96] concluded that PIs can inhibit fungal growth by inhibiting endogenous trypsin that is essential for chitin synthase, thus blocking chitin synthesis in fungal cell walls [97]. Since the role of specific microbial proteases in pathogenicity is unclear, the effects of plant PIs on the activity of these enzymes need further study.

**Table 1:** Main families of botany-derived antimicrobial peptides (BAMPs) and their modes of action.

Family	Representative Peptide	Sources	Mode of Action	References
Defensin (PR12)	DmAMP1	*Dahlia merkii*	DmAMP1 binds to M(IP)2C in the membrane, resulting in potassium efflux, calcium uptake, membrane permeability change, and CWI pathway activation.	[25,28,29]
	RsAFP2	*Raphanus sativa*	RsAFP2 binds to GlcCer in the cell wall and plasma membrane, resulting in ROS production, apoptosis, ion fluxes, and CWI pathway activation.	[30,31]
	MsDef1	*Medicago sativa*	MsDef1 interacts with GlcCer in the cell wall and membrane, resulting in the activation of MAPK cascade in the CWI pathway, and disruption of Ca^2+^ signaling and homeostasis, contributing to fungal cell death.	[32,33,98]
	MtDef4	*Mendicago trucatula*	MtDef4 binds to PA and is internalized into the fungal cell, resulting in the disruption of Ca^2+^ signaling and homeostasis in a different way to MsDef1. An interaction with unknown intracellular targets has been proposed.	[34]
	NaD1	*Nicotiana alata*	NaD1 binds to PI(4,5)P2 and dimerizes in the membrane. The dimer is internalized into the cytoplasm and interacts with intracellular targets to trigger the ROS and NO production.	[33,35]
	HsAFP1	*Heuchera sanguinea*	HsAFP1 binds to the fungal cell wall and plasma membrane via loop 5. It moves into the cytoplasm and targets mitochondria, produces ROS, and induces programmed cell death.	[36]
	Psd1	*Pisum sativum*	Psd1 binds to GlcCer in the plasma membrane. It moves to the cytoplasm and interacts with cyclin F in the nucleus, which results in cell cycle arrest and fungal cell death.	[37]
PR1	PR1a	*Nicotiana tabacum*	PR1 binds to sterol and sequesters it from pathogens. It inhibits programmed cell death at the pathogen infection sites, and induces the expression of host defense genes by releasing CAPE1 peptide.	[43,45,46,47]
	P14c	*Solanum lycopersicum*		
Thionins (PR13)	α_1_-purothionin β-purothionin	*Triticum aestivum*	Upon application of purothionin, there is a depolarization of the membrane and Ca^2+^ ion permeability increases. β-purothionin interacts with dimyristoyl-phosphatidylglycerol, and inhibits protein kinase C.	[59,99]
	Thionin	*Pyrularia pubera*	Thionin leads to membrane depolarization, influx of exogenous Ca^2+^, and activation of PLA2 and adenylate cyclase.	[62]
	viscotoxins A3 viscotoxins B	*Viscum album*	Viscotoxins directly interact with DNA and RNA, interfering with nucleic acid synthesis.	[100]
Lipid transfer proteins (LTPs, PR14)	Ca-LTP1	*Capsicum annuum*	Ca-LTP1 penetrates the plasma membrane and causes morphological damage, accompanied by pseudo-mycelia formation.	[77]
	Ha-AP10	*Helianthus annuus*	Ha-AP10 interacts with phospholipids and produces a direct cytotoxic effect on fungal cells mediated by membrane permeabilization.	[78]
Proteinase inhibitor (PIs, PR6)	HyPep	*Capsicum annuum*	HyPep inhibits α-amylase and serine proteinases, and causes cell aggregation and pseudo-mycelia formation.	[94]

## 3. Antimicrobial Activity of BAMPs against Targeted Organisms and Cancer Cells

In addition to defending against plant disease, BAMPs are also potential candidates for the treatment of human diseases. Here, we mainly focus on the antifungal, antiviral, and anticancer activity of BAMPs in plants and humans.

### 3.1. Antifungal Activity

Pathogenic fungi are considered a greater threat to plant and animal biodiversity than other taxonomic classes [101]. As part of the intrinsic immune system, BAMPs have great potential to be developed as novel antifungal agents due to their broad-spectrum activity, selective targeting, multiple mechanisms of action, and limited cell cytotoxicity [7,102]. Elucidating the functional mechanism of BAMPs is key to uncovering their application potential and developing new therapeutic approaches.

**Figure 2:**
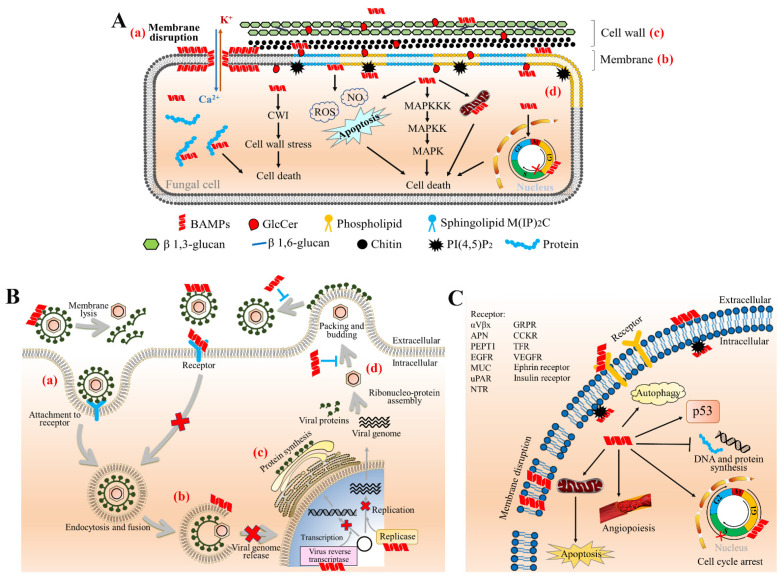
The possible mechanism of BAMPs in antifungal, antiviral, and anticancer activities. (**A**) The antifungal activity of BAMPs. The antifungal mechanisms of BAMPs are divided into the following categories: (**a**) BAMPs destroy the membrane permeability through electrostatic interaction with the membrane surface. (**b**) BAMPs affect membrane structure or in vivo signaling pathways by interacting with lipids such as phospholipids and sphingolipids on fungal membranes. (**c**) BAMPs interact with fungal cell wall components such as glucan and chitin, thus inhibiting cell wall formation and pathogen growth. (**d**) BAMPs act on intracellular targets and participate in cellular signaling pathways, including ROS production, programmed cell death, cell cycle arrest, autophagy, the CWI (cell wall integrity) pathway, the MAPK pathway, and so on. (**B**) The antiviral activity of BAMPs. The replication cycle of the virus during infection is roughly divided into four steps: (**a**) By recognition and binding to receptors, viruses attach to and fuse with the host membrane. (**b**) Virus uncoating and release of nucleic acid. (**c**) Viral genome replication and protein synthesis. (**d**) Assembly and release of virus particles. During virus replication, BAMPs inhibit virus proliferation and transmission by destroying virus envelope, inhibiting the interaction between capsid proteins with host cell surface receptors, blocking the expression of virus gene, and preventing the assembly and release of virus particles. (**C**) The anticancer activity of BAMPs. Obvious differences exist in normal and tumor cells, including the charge and receptors on the membrane. On the one hand, BAMPs can especially kill cancer through membrane dissolution; on the other hand, BAMPs regulate angiogenesis, apoptosis, autophagy, cell cycle, and other biological processes which are critical to tumor proliferation and migration after binding to receptors.

The mechanisms underlying the antifungal action of BAMPs can be grouped into the following categories (Figure 2A): (1) BAMPs interact with the fungal cell surface. Through electrostatic action, BAMPs adsorb onto the membrane surface of pathogens, and form a central lumen in the membrane through barrel-stave, carpet, or toroidal pore mechanisms, which induces lipid bending and eventually leads to pore formation [7]. (2) BAMPs bind to the components on the fungal cell membrane. The cell membrane is mainly composed of sterols, phospholipids, and sphingolipids. Based on the polar head groups, phospholipids can be further divided into PA, phosphatidylcholine, phosphatidylethanolamine (PE), phosphatidylglycerol, phosphatidylserine (PS), and inosinephosphatidylinositides, while sphingolipids are divided into sphingomyelin and glycosphingolipids. Many BAMPs can interact with cell membrane components, such as phospholipids and sphingolipids. For example, NaD1 kills fungal cells by binding to PA via the 39th arginine [103]. GlcCer, the most common glycosphingolipid in fungi [104,105], also plays an important role in the antifungal activity of BAMPs, including MsDef1, Psd1, Psd2, PvD1, Sd5, and RsAFP2. Interestingly, although most sphingolipids are not essential for cell survival, they are key regulators of the pathogenicity of various fungi and are essential components needed for infection in vivo [106]. (3) BAMPs interact with the fungal cell wall. Fungal cell walls are mainly composed of glucan, chitin, and glycosylated proteins [107]. GlcCer is also an important component of fungal cell walls [108]. A variety of BAMPs are able to bind to major cell wall components, such as RsAFP2 targeting GlcCer, and hevein-like peptides binding chitin, thereby inhibiting cell wall formation and pathogenic fungal growth [109]. Notably, BAMPs and human-derived AMPs such as histatin 5 and neutrophil defensin 1 (HNP-1) have a similar mechanism of inhibiting cell wall biosynthesis [110]. (4) BAMPs act on intracellular targets and participate in cellular signaling pathways. The antifungal mode of BAMPs in vivo mainly includes the induction of endogenous ROS production and programmed cell death, mitochondrial dysfunction, adenosine triphosphate (ATP) efflux, cell cycle disorder, cation homeostasis disruption, autophagy induction, and vesicular dysfunction [7]. BAMPs such as HsAFP1 and NaD1 are capable of internalization within the fungal cytoplasm through endocytosis to exert their antifungal effects [7]. Notably, the internalization process of BAMPs is often species-specific. For example, OefDef1.1 transfers to the cytoplasm at the plant germling and pathogenic conidia stages in *F. oxysporum*, whereas the internalization only occurs at the germ development stage in *B. cinerea* [111]. Furthermore, BAMP internalization is not indispensable for the induction of intracellular mechanisms. For example, RsAFP2 does not need to be internalized within *C. albicans* to induce ROS production and programmed cell death [31].

### 3.2. Antiviral Activity

BAMPs can control viral infection by disrupting viral envelope structures, blocking the interaction of viruses with host cells, and inhibiting viral replication (Table 2). StPIP1, a pathogen-associated molecular pattern (PAMP)-inducible peptide in potato, triggers plant defense responses against potato Y virus (PVY) through inducing ROS production, callose deposition, and defense-related gene expression during compatible interaction with PVY [112]. Peptides A22 and A64 interfere with viral replication by binding to the origin of replication loop structure (OriRep) of tomato golden mosaic virus (TGMV) [113]. Co-expression of these two peptides in tomato plants infected with tomato yellow leaf curl virus (TYLCV) or tobacco mottle virus (ToMoV) effectively reduces the disease symptoms [113]. Consistent with the action mechanism of A22 and A64, the AmPep1 peptide obtained from the globulin of *Amaranthus hypochondriacus* seed highly binds to the OriRep of TYLCV and pepper yellow vein virus (PHYVV), inhibiting viral replication and alleviating disease symptoms in *Nicotiana benthamiana* [114]. This was the first report of direct exogenous expression of a peptide for controlling plant DNA virus. Since then, Rudolph et al. [115] improved tobacco resistance to tomato chlorotic spot virus (TCSV), groundnut ringspot virus (GRSV), chrysanthemum stem necrosis virus (CSNV), impatiens necrotic spot virus (INSV), iris yellow spot virus (IYSV), physalis severe mottle virus (PSMV), and watermelon silver mottle virus (WSMV), by transgenically expressing the dominant transacting peptides of 29 amino acids that strongly interact with the nucleocapsid proteins of different viruses.

Plants are also an excellent source of antiviral peptides for human viruses. For example, a 9 kDa nonspecific LTP (NTP) isolated from *Narcissus tazetta* var. *chinensis* L. inhibits the proliferation of influenza A (H1N1) virus by blocking the ceramidase on the viral envelope [16]. Further, NTP prevents respiratory syncytial virus (RSV) from entering host cells and interferes with RSV transmission by binding to viral glycoproteins or inhibiting other events in viral replication or assembly [16]. Moreover, ginkbilobin isolated from ginkgo seeds, ascalin from *Allium ascalonicum* bulbs, lunatusin from *Phaseolus lunatus* L. seeds, and vulgarinin from *Phaseolus vulgaris* seeds can inhibit HIV-1 proliferation by inhibiting HIV-1 reverse transcriptase activity [116,117,118,119]. Meliacine (MA), a peptide isolated from the leaves of the *Melia azedarach* L., inhibits the proliferation of foot-and-mouth disease virus (FMDV) in BHK-21 cells by blocking the uncoating process of the virus through inhibiting vesicular acidification [120]. Pep-RTYM, a novel active peptide isolated from the Asian medicinal plant *Acacia catechu*, demonstrates broad antiviral activity against four serotypes of dengue virus (DENV) in the early stages of viral infection [121,122]. By binding to DENV particles, Pep-RTYM prevents interaction of the virus with cellular receptors and the subsequent release of nucleic acids, without apparent cytotoxicity [122].

Besides BAMPs, some proteins also function in plant antiviral resistance and have shown great potential for plant protection applications. JAX1, a jacalin-type lignan-like lectin protein identified from Bay-0 ecotype *A. thaliana*, significantly increases the plant’s resistance to potato X virus (Potexvirus), and was further found to interact with the RNA-dependent RNA polymerase (RdRp) of Potexvirus, inhibiting its replication activity and thus blocking viral infection [123,124]. Beclin1 (ATG6) is an autophagy core protein that acts as a selective autophagy receptor, targeting viral replicase (a nuclear inclusion ‘b’ protein, NIb) and mediating ATG8a-dependent selective autophagy [125]. Additionally, Beclin1 is able to interact with viral NIb and inhibit its RdRp activity in an autophagy-independent manner [125]. Cyclophilin Cpr7p, a molecular chaperone in plants and animals, strongly inhibits viral RNA recruitment, the assembly of viral replicase complexes, and viral RNA synthesis during tomato bushy stunt virus (TBSV) replication [126]. Using the active regions of these antiviral proteins to prepare specific antiviral peptides would provide new resources for broad-spectrum disease control.

Collectively, antiviral BAMPs may control viral infection and proliferation by (Figure 2B): (1) Disrupting the viral envelope, resulting in membrane destabilization and disruption, thus inhibiting the virus’ ability to infect host cells [127,128,129]. (2) Blocking viral binding to host cells by competitively binding to viral capsid proteins or host cell surface receptors, thereby preventing viral uncoating and genome release into host cells [130,131,132,133,134,135]. (3) Crossing the host cell membrane into the cytoplasm or nucleus to elicit the host defense system against the virus or regulating cellular pathways to block viral gene expression, thereby inhibiting viral replication [118,119,136]. (4) Targeting structural proteins to inhibit the assembly of viral particles, enhance host phagocytosis, etc., thereby preventing viral replication and transmission [127]. Integrating BAMPs resistance against plant and animal viruses and exploring their target proteins will provide new ideas and approaches for the development of antiviral peptide drugs for agricultural and medical use.

### 3.3. Anticancer Activity

BAMPs are important members of the anticancer drug family (Table 3). The most well-known anticancer BAMP is lunasin, a small peptide consisting of 43 amino acid residues from the 2S albumin in soybean seed, which contains three functional domains: the chromosome-targeting domain, the cell adhesion (Arg-Gly-Asp, RGD) domain, and the cysteine tail that binds histones H3 and H4. By targeting cellular chromatin, this small peptide acts on the highly basic region of the N-terminal end of histones in the centromere, disrupting the normal formation of the mitotic complex, blocking normal mitosis, and ultimately leading to cancer cell death [137]. Now, lunasin has been identified in a variety of plants, including barley, wheat, loblolly, quinoa, and oats [138,139]. In addition, cycloviolacin O2 (CyO2), a cyclic peptide isolated from *Viola odorata*, is a promising anticancer drug that causes necrosis of human lymphoma cells by disrupting cell membranes and is selectively toxic to tumor cells relative to normal cells [140]. The cyclic peptide MCo-PMI, obtained by engineering processing MCoTI-I, inhibits prostate tumor growth by activating the p53 tumor suppressor pathway [141]. The cyclic peptide HB7 from *Hedyotis biflora* significantly inhibits tumor proliferation and migration in an in vivo xenograft model [142]. The action mechanism of these cyclic peptides may be related to their ability to target and disrupt cell membranes; therefore, a better understanding of the membrane specificity of cancer cells will help design novel drugs based on the cyclic peptide framework, and allow specific peptide drugs to target different cell types. Additionally, NaD1 has been shown to inhibit the proliferation of monocytic lymphoma through direct binding to the phosphatidylinositol 4,5-bisphosphate (PIP2) on the plasma membrane [143]. Viscotoxin B2 inhibits rat osteogenic sarcoma through membrane lysis [144], and ligatoxin B inhibits the proliferation of lymphoma and adenocarcinoma cells through inhibiting nucleic acid and protein synthesis [145]. Relative to other BAMPs, increasing evidence supports that LTPs play an important role in tumor progression and metastasis. Phosphatidylinositol, sphingolipids, and fatty acids act as second messengers in key signaling pathways that control cell survival, proliferation, and migration. LTPs such as NTP [16] mediate cancer-associated signaling cascades by regulating the distribution of lipids within the cell membrane, thereby inhibiting tumor cell infiltration and metastasis [146]. There are also many other antitumor BAMPs of which the action mechanisms are unclear, such as phoratoxins C–F [147], Thi2.1 [148], sesquin [149], limenin [150], and coccinin [151].

Besides BAMPs, other small peptides demonstrate anticancer activity in plants (Table 3). For example, Cn-AMP1 derived from *Cocos nucifera* can reduce cancer cell viability without causing hemolysis [152] and Cr-ACP isolated from *Cycas revoluta* arrests the Hep2 cell cycle in the G0–G1 phase [153]. Further, the cyclic heptapeptide cherimolacyclopeptide C isolated from *Annona cherimola* seeds displays in vitro cytotoxicity against KB cells [154], while a cell cycle-inhibiting octapeptide cyclosaplin purified from *Santalum album* L. inhibits breast cancer cell proliferation in a dose- and time-dependent manner [155]. Moreover, the cyclic peptides Poca A and B isolated from *Pombalia calceolaria* roots can inhibit breast cancer cell migration, which are inactive at less than 1 μM, and are toxic at higher concentrations [156]. IbACP, a small peptide of 16 amino acid residues obtained from the leaves of sweet potato, is able to rapidly alkalinize cell tissues and induce apoptosis in tumor cells through a mitochondria-dependent pathway [157].

Taken together, the above studies indicate that BAMPs can kill cancer cells by membrane lysis (Figure 2C) [158]. Owing to the composition of phosphatidylserine, *O*-glycosylated mucin, sialylated gangliosides, and heparan sulfate on the membrane surface of cancer cells, the membrane has a negative charge, which is in contrast to normal mammalian cell membranes [159]. Thus, positively charged BAMPs can selectively target cancer cells. However, the non-membrane solubilizing activity of BAMPs is involved in regulating processes such as angiogenesis, apoptosis, autophagy, and cell cycle that are critical for tumor proliferation and migration (Figure 1C) [160]. Normal and tumor cells differ in many ways, such as the presence of specific receptors such as integrin αvβx, aminopeptidase APN, peptide transporter protein PEPT1, and epidermal growth factor receptor EGFR on the surface of cancer cells. In addition to interfering with membrane permeability through electrostatic effects, do BAMPs bind specifically to cancer cell surface receptors and kill cancer cells by inducing host immune responses or by entering intracellular to target signaling pathways? Exogenous proteins have been shown to enter cells by integrin receptor-mediated means [161]. Therefore, ideas for novel anticancer drugs may be obtained by screening AMPs in medicinally valuable plants, animals, and microorganisms focusing on cancer cell surface receptors.

**Table 3:** Representative botany-derived antimicrobial peptides (BAMPs) and some other small peptides with anticancer activity and their modes of action.

Classification	Representative Peptide	Anticancer Activity	Mode of Action	References
BAMPs	Lunasin	Skin, colon, prostate, and breast cancers	Lunasin binds directly to deacetylated histones, inhibits acetylation, and turns off the transcription.	[137]
	Cycloviolacin O2 (CyO2)	Breast cancer and lymphoma cells	CyO2 causes tumor cell death by membrane permeabilization.	[140]
	MCo-PMI	Adenocarcinoma	MCo-PMI inhibits tumor proliferation by activating the p53 tumor suppressor pathway.	[141]
	HB7	Pancreatic cancer	HB7 inhibits the proliferation and migration of tumors by membrane permeabilization.	[142]
	NaD1	Monocyte lymphoma	NaD1 inhibits the proliferation of monocyte lymphoma by directly binding to the plasma membrane phosphatidylinositol 4,5-diphosphate.	[143]
	Viscotoxin B2	Osteogenic sarcoma	Viscotoxin B2 inhibits tumor cells by membrane lysis.	[144]
	Ligatoxin B	Lymphoma and adenocarcinoma	Ligatoxin B inhibits the proliferation of tumor cells by inhibiting nucleic acid and protein synthesis.	[145]
	NTP	Promyelocytic leukemia cells (HL-60)	NTP mediates cancer-related signal transduction cascades by regulating the distribution of lipids in cell membranes, thereby inhibiting tumor cell invasion and metastasis.	[16,146]
	Phoratoxins C-F	Different types of solid tumor cells and hematologic tumors	Inhibiting tumor proliferation, while the mechanism of action is unknown.	[147]
	Thi2.1	Tumor cells McF-7, A549, and HeLa		[148]
	Sesquin	McF-7 and leukemia M1 cells		[149]
	Limenin	Leukemia cells		[150]
	Purple pole defensin	HepG2, McF-7, and HT-29 cells		[162]
	Coccinin	HL60 and L1210 cells		[151]
Other small peptides	Cn-AMP1	Caco-2 cells	Cn-AMP1 reduces cancer cell viability without causing hemolysis.	[152]
	Cr-ACP	Hep2 cells	Cr-ACP induces cell cycle arrest in G0–G1 phase.	[153]
	Cherimolacyclopeptide C	KB cells	Cherimolacyclopeptide C shows in vitro cytotoxicity to KB cells.	[154]
	Cyclosaplin	Breast cancer	Cyclosaplin inhibits cancer cell proliferation in a dose- and time-dependent manner.	[155]
	Poca A and B	Breast cancer	Poca inhibits cancer cell migration.	[156]
	IbACP	Panc-1, a pancreatic cancer line	IbACP regulates cellular proliferation by inducing and promoting apoptosis through the mitochondrial apoptotic pathway.	[157]
	GLTSK	HCT116, human colorectal cancer cells	GLTSK decreases angiotensin II-dependent proliferation in HCT116 through the blockade of the renin-angiotensin system.	[163]

## 4. Applications of BAMPs for Agricultural Purposes

The in vitro and in vivo activities of many BAMPs are well-known and show clear potential for agricultural purposes. Plants expressing exogenous BAMP variants, synthetic BAMPs, and isogenous BAMPs are capable of producing resistance to a variety of pathogens [164,165]. Particularly, defensins have been heterologously expressed in many economically important crops to enhance resistance to pathogenic fungi. The radish defensin Rs-AFP2 was the first heterologous BAMP to be expressed in other plants, inducing protection against *Alternaria longipes* in tobacco and tomato [13], and against different fungi in wheat [14]. Other BAMPs also have been used to induce resistance in different plants, such as overexpression of Pn-AMP in tobacco and tomato enhances their resistance to *Phytophthora nicotianae* and *Phytophthora capsici*, respectively [166]. Further, overexpression of the barley LTP gene in *A. thaliana* enhances its resistance to *Pseudomonas syringae* and *Staphylococcus griseus* [167]. Compared with resistance enhancement by overexpression of a single gene, co-expression of multiple genes could incur stronger resistance. For example, co-expression of the defensins Dm-AMP1 and Rs-AFP2 in rice results in plants with stronger antifungal activity than the expression of either defensin alone [168]. In addition to their antimicrobial activity, BAMPs are able to promote plant growth and optimize crop traits. Therefore, combining their antimicrobial ability with growth-promoting effects can better control disease, improve crop yield, and ensure food security. Additionally, many non-plant AMPs also play an important role in plant protection in agriculture. For example, cecropin A and B from *Hyalophora Cecropia* are expressed in rice and tomato, enhancing the resistance of these crops to bacterial and fungal diseases [169,170,171]. The undecapeptide BP100 and its derivatives, which were identified from a library of synthetic cecropin A-melittin hybrids, can also improve the anti-pathogen activity of plants [172,173]. Unfortunately, due to their limited stability and activity conditions, BAMPs and non-plant AMPs have not yet been directly prepared as commercial biofungicides.

**Figure 3:**
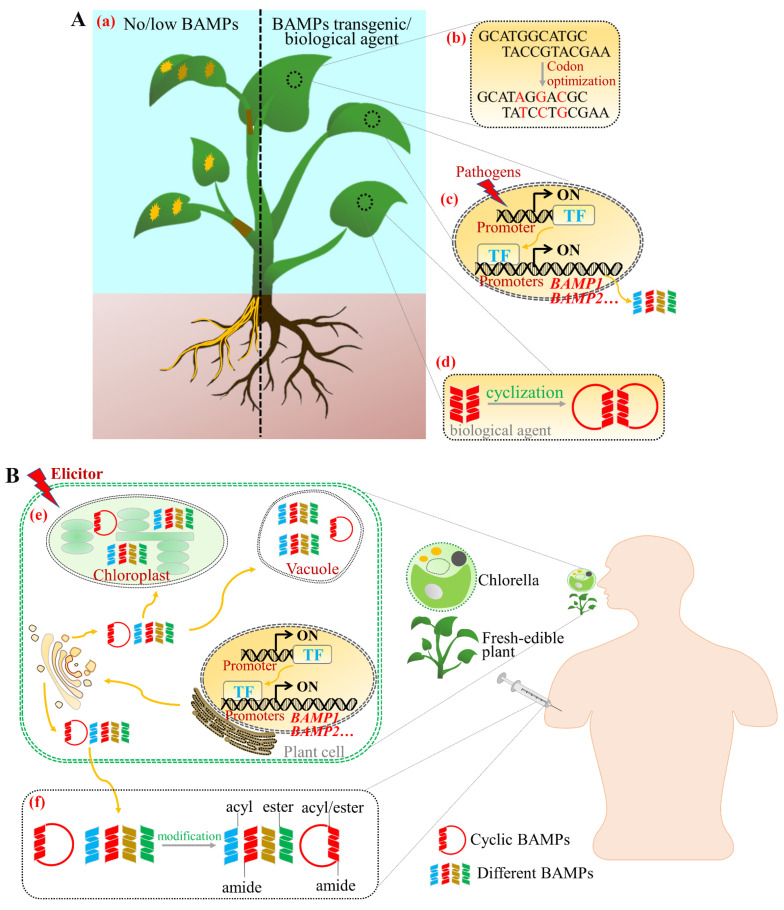
The proposed application strategies of BAMPs for agricultural purposes and human health. (**A**) The application of BAMPs for plant disease control. Plants without or with low BAMPs are vulnerable to fungi and viruses (**a**), while transgenic expression of BAMPs in vivo or application of BAMPs’ biological agents in vitro can enhance the resistance of host plants to pathogens. Codon optimization (**b**) and multigene-induced expression (**c**) can elevate *BAMPs* expression and broaden host resistance to pathogens. Furthermore, modification of BAMPs in vitro, such as cyclization, can enhance their stability and promote antimicrobial activity, which can be directly used as a biological agent in the future (**d**). (**B**) The application of BAMPs for human health. Using fresh-edible plants or chlorella as a bioreactor to prepare oral BAMPs drugs can not only break through the restriction of intravenous injection, but also greatly increase the yield and decrease the cost (**e**). On the one hand, BAMPs can be biosynthesized in organelles such as vacuoles and chloroplasts to protect them from being degraded by digestive enzymes during oral administration. On the other hand, BAMPs can be directly secreted in the fermentation broth and be prepared for intravenous injection when chlorella is used as a bioreactor. Additionally, N- and/or C-terminus modification of BAMPs can increase their stability and their ability to pass through the biological membrane (**f**).

The rational, efficient, and innovative use of BAMPs will have a multiplier effect on the disease prevention and control. In summary, the following strategies are proposed (Figure 3A): (1) Exploring the expression regulation mode of BAMPs and optimizing BAMPs codons to increase their expression and activity. (2) Excluding the toxic effects of BAMP accumulation on host plants by using pathogens to induce high expression of BAMPs with high activity, multiple mechanisms of action, and low toxicity. (3) Inducing synergistic expression of BAMPs from different sources, with variable mechanisms of action to expand the range and variability of host resistance to pathogens. (4) Modifying BAMPs in vitro to enhance their stability and improve disease resistance for preparing them into biological agents for direct external application. Overall, the use of BAMPs for both molecular breeding and direct external application can reduce the use of chemical pesticides, control crop losses, and ensure food security. Combining multiple biotechnologies can better expand their application potential.

## 5. Applications of BAMPs for Human Health

BAMPs have emerged as important novel candidates for the treatment of various human infections due to their high efficiency, specific selectivity, broad range of targets, high permeability in tissues, low immunogenicity, low toxicity, and tissue accumulation. In addition to antimicrobial effects, some BAMPs have shown promise as anticancer agents, as their activity against target cells and/or induced immune responses can effectively control infections and reduce tumorigenesis [10]. Further, BAMPs have shown promising therapeutic effects in combination with conventional therapies [10]. The most well-studied BAMP, lunasin [137], has become the reference and standard for elucidating the anticancer mechanism of BAMPs, and provides a viable candidate for drug development. In addition, the reported anticancer mechanisms of BAMPs, such as viscotoxin B2 [144], ligatoxin B [145], NaD1 [143], CyO2 [140], MCo-PMI [141], and NTP [16], have indicated their potential to be developed as anticancer drugs. The numerous advantages of BAMPs have successfully attracted the attention of the pharmaceutical industry [174]. Currently, some BAMPs and their derivatives are in various clinical trial stages, such as Brilacidin (defensin mimetic), Surotomycin (cyclic lipopeptide), PAC-113 (12 amino acid antimicrobial peptide), and HB1275 (lipohexapeptide) [175]. Like other peptides of natural origin, BAMPs mostly exist at very low concentrations, and would need to be produced in large quantities for pharmaceutical or biological applications [10]. Although the development process is similar to that of traditional small molecule drugs, peptide drugs require unique process designs, preparation methods, structure confirmation, and production equipment. In particular, the large-scale commercial production of high-purity antimicrobial peptides is a practical demand for the treatment of diseases. The lack of a suitable manufacturing platform in terms of product yield, cost, and purity is a barrier to the medical use of BAMPs. Recent advances in biotechnology allow plants to be employed as bioreactors for BAMPs production, as mentioned above [176]. In addition, non-plant AMPs can also be expressed in plants for their use in a global health strategy. For example, retrocyclin-101 (RC101) and protegrin-1 (PG1) are two important antimicrobial peptides that can be used to treat bacterial and/or viral infections, especially those caused by HIV-1 or sexually transmitted bacteria. Lee et al. used plant molecular farming to achieve stable expression of these two AMPs in tobacco, which accounted for 38% and 26% of total soluble protein of chloroplast, respectively [177]. Similarly, lactostatin is an anti-hypercholesterolemic peptide derived from β-lactoglobulin in cow’s milk. Cabanos et al. realized that the expression of lactostatin in rice, and its content in dry seeds, reached 2 mg/g, which has potential clinical application value as an anti-high-cholesterol peptide drug [178]. Therefore, the establishment of transgenic plants expressing bioactive BAMPs and non-plant AMPs is a promising strategy for the production of therapeutic AMPs. In addition, peptides have a short half-life and are easily broken down by enzymes in the body, making them difficult to absorb through the digestive system. Accordingly, peptide drugs are often administered by injection.

Based on the limitations of peptide drug production and clinical delivery methods, the following solutions are proposed (Figure 3B): (1) Prioritizing cyclic peptides or cyclizing linear peptides with high antimicrobial activity and anticancer activity, since the head-to-tail cyclic backbone and the cysteine binding motif of cyclic peptides make them more stable and resistant to thermal or enzymatic degradation [179]. Additionally, the cyclization of linear peptides leads to a reduction in their conformational flexibility or actually decreases the number of hydrogen bonds formed by the peptide, thus increasing their ability to pass through biological membranes and their resistance to endopeptidases and exopeptidases [180]. (2) Using fresh-edible plants as bioreactors and BAMPs with medicinal value as a starting point, then, modify the N-terminus of BAMPs by compartmentalization and localization to allow targeted biosynthesis of BAMPs in organelles such as vesicles and chloroplasts, thereby adding a protective layer and reducing their degradation by the digestive tract during consumption. (3) Combined with synthetic biology, scaling up production of medicinal BAMPs using *Chlorella pyrenoidosa* as a bioreactor, which could be photoautotrophic and heterotrophic. *C. pyrenoidosa* is a single-celled algae that is rich in nutrients and protein content (more than 50% of dry weight), making it a new resource food with health value. Due to its fast growth rate, simple nutritional requirements, low cost, and that it can be cultivated via heterotrophic fermentation on a large scale, *C. pyrenoidosa* is an ideal chassis for biosynthesis. On the one hand, using *C. pyrenoidosa* as a chassis to synthesize BAMPs can directly treat diseases through oral administration. On the other hand, BAMPs could be synthesized and secreted outside the cells, enabling extraction from the fermentation broth and subsequent modification to enhance their stability for oral or intravenous administration. (4) Modifying the N- and/or C-terminus of the peptide sequence through N-acylation, N-esterification, or C-amidation using biotechnology to increase the stability of BAMPs and enhance their ability to pass through biological membranes [180].

## 6. Outlook

Researchers have gained a profound understanding of BAMPs in recent decades. To date, they have isolated and identified hundreds of BAMPs, analyzed their functions against plant pathogens, and elucidated the mechanisms underlying the antimicrobial action of some BAMPs. However, there remains a big knowledge gap for the development of BAMPs into anti/fungicidal agents for application in plant protection. Firstly, the mechanism of action remains unclear. Some studies have reported that BAMPs interact with the cell membrane and cell wall components of pathogens, causing membrane perforation and leakage of cell contents, which achieves antimicrobial efficacy. However, the detailed process of membrane perforation caused by BAMPs requires further study. Further, some studies have reported that BAMPs can be internalized into the cytoplasm and interact with intracellular targets to mediate cellular signaling pathways, leading to apoptosis of pathogenic cells. However, it is unknown how BAMPs are internalized into cells, which targets they interact with, and how they mediate cellular signaling in pathogen cells. The lag in mechanistic studies has hindered our in-depth understanding of BAMPs. Secondly, multiple BAMPs are upregulated to participate together in the immune response when plants are infected by pathogens. In practical applications, BAMPs’ synergism may be required to achieve optimal efficacy. Finally, external application of BAMPs faces limitations such as short half-lives, weaker resistance, and harsh activity conditions. Therefore, enhancing the stability of BAMPs is an inescapable problem that must be resolved prior to their industrial application.

The field of peptide therapeutics is rapidly growing. To date, more than 80 peptide drugs have been approved by the FDA or the EU EMA, and dozens of AMPs are currently being evaluated in clinical trials. Although some studies report BAMP efficacy in cancer inhibition, as well as inhibition of viral replication and propagation, no drugs developed with a BAMP backbone have yet to be marketed. Lunasin is one of the more thoroughly studied BAMPs, which not only targets the chromatin in cells, but also disrupts the formation of the mitotic complex in the N-terminal region of histones and blocks normal mitosis. Additionally, lunasin is able to target tumor cell integrin receptors specifically through its RGD domain and mediate the integrin signaling pathway, thereby inhibiting tumor cell proliferation. Most importantly, lunasin is resistant to digestion and can be absorbed directly after oral administration to reach specific tissue sites, which provides a reference for the development of therapeutic foods and oral BAMP-derived drugs, and provides insight for basic research on BAMPs. Is it possible for BAMPs to interfere with signaling pathways by targeting specific tumor cell surface receptors, while being internalized intracellularly, to interact with target proteins and thus induce apoptosis or other biological process in cancer cells? Moreover, due to their short half-lives and instability, peptides are currently administered locally by intravenous injection. Developing BAMPs for direct therapeutic feeding or oral administration would be a meaningful and promising alternative. Based on synthetic biotechnology, whole edible plants or edible microalgae can be used as bioreactors, and compartmentalized synthesis and amino acid modification can be used to synthesize BAMPs, thus optimizing the structural stability of BAMPs and solving scale-up issues for the industrial production of BAMPs.

Currently, the global market for peptide drugs has significant growth potential. Future in-depth research addressing the aforementioned limitations of BAMPs will enable their successful application in agriculture and human health. Due to the advantages of high yield, high quality, homogeneity, and post-translational modifications such as glycosylation and disulfide bond formation that are critical to BAMPs’ activity in plant bioreactors, a plant-based BAMPs’ production platform is more promising than other biological systems [176]. It is believed that plant molecular farms can play a greater role in the production of BAMPs if the yield and stability of the products can be further solved to meet the needs of the market, as well as the safety, quality, and effectiveness.

## Figures and Tables

**Table 2:** Representative botany-derived antimicrobial peptides (BAMPs) with antiviral activity and their modes of action.

Source of Virus	Representative Peptide	Antiviral Activity	Mode of Action	References
Plant virus	StPIP1	Potato Y virus (PVY)	StPIP1 induces the ROS production, callose deposition, and expression of defense-related genes in plants.	[112]
	A22 and A64	Tomato Golden Mosaic virus (TGMV)	Peptides interfere with virus replication by binding to the replication origin sequence (OriRep).	[113]
	AmPep1	Tomato yellow leaf curl virus (TYLCV) Pepper yellow vein virus (PHYVV)		[114]
	Dominant transacting peptide	Tomato chlorotic spot virus (TCSV) Groundnut ring spot virus (GRSV) Chrysanthemum stem necrotic virus (CSNV) Impatiens necrotic spot virus (INSV) Iris macular spot virus (IYSV) Physalis severe mottle virus (PSMV) Watermelon silver mottle virus (WSMV)	The peptide interacts with the nucleocapsid proteins (N) of different tospoviruses and induces host immune responses.	[115]
Animal virus	NTP	Influenza A virus (H1N1)	NTP inhibits virus proliferation by blocking the neuramidase on the virus envelope, and inhibits the cytopathic effect induced by H1N1.	[16]
		Respiratory syncytial virus (RSV)	NTP prevents RSV entry into host cells and proliferation by binding to the viral glycoproteins or inhibiting viral replication and assembly.	
	Ginkbilobin	HIV-1	Peptides suppress the activity of HIV-1 reverse transcriptase.	[116,117,118,119]
	Ascalin			
	Lunatusin			
	Vulgarinin			
	Meliacine	Foot-and-mouth disease virus (FMDV)	Meliacine prevents the hulling process of FMDV by inhibiting vacuolar acidification, and thus restrains the virus proliferation.	[120]
	Pep-RTYM	Dengue virus (DENV)	Pep-RTYM binds to the DENV particles to prevent viral interaction with host cell receptors and the subsequent nucleic acid release.	[121,122]

## Data Availability

Not applicable.
